# *APOE*‐mediated sex differences in microvascular pathology and AD‐associated proteinopathies in the medial temporal lobe

**DOI:** 10.1002/alz.71206

**Published:** 2026-03-14

**Authors:** Francesco Bax, Jan Oltmer, Corinne A. Auger, Anna Bonkhoff, Gillian T. Coughlan, Alexandra Melloni, Methasit Jaisa‐Aad, Jean Augustinack, Alberto Serrano‐Pozo, Matthew P. Frosch, Steven M. Greenberg, Teresa Gomez‐Isla, Bradley T. Hyman, Susanne J. van Veluw, Valentina Perosa

**Affiliations:** ^1^ J. Philip Kistler Stroke Research Center Department of Neurology Harvard Medical School Massachusetts General Hospital Boston Massachusetts USA; ^2^ Clinical Neurology Unit Department of Head, Neck and Neurosciences Udine University Hospital Udine Italy; ^3^ BetterDoc GmbH Cologne Germany; ^4^ MassGeneral Institute for Neurodegenerative Disease Massachusetts General Hospital Charlestown Massachusetts USA; ^5^ Department of Neurology Massachusetts General Hospital Harvard Medical School Boston Massachusetts USA; ^6^ C.S. Kubik Laboratory for Neuropathology Neuropathology Service Harvard Medical School Massachusetts General Hospital Boston Massachusetts USA; ^7^ British Heart Foundation ‐ UK Dementia Research Institute Centre for Vascular Dementia Research The University of Edinburgh Edinburgh UK

**Keywords:** Aβ plaques, Alzheimer's disease, *APOE*, arteriolosclerosis, cerebral amyloid angiopathy, deep‐learning, medial temporal lobe, microvascular pathology, mixed‐pathologies, neuropathology, pTDP‐43, sex differences, tau tangles

## Abstract

**INTRODUCTION:**

Cerebral small vessel disease (CSVD) contributes to the development of Alzheimer's disease (AD) dementia and co‐occurs with AD‐associated proteinopathies. However, how sex modulates the interaction between CSVD and AD‐associated proteinopathies in the medial temporal lobe (MTL) remains unclear.

**METHODS:**

One hundred fifty‐two autopsy cases from the Massachusetts Alzheimer's Disease Research Center were included. Deep‐learning and semiquantitative scores were applied to MTL histological sections to obtain quantitative measures of proteinopathies and CSVD (cerebral amyloid angiopathy [CAA] and arteriolosclerosis). The effect of sex on AD‐associated proteinopathies and the interaction between sex, CSVD, and apolipoprotein E (*APOE*) genotype were analyzed using linear mixed‐effect models.

**RESULTS:**

In women, higher CAA burden was associated with lower amyloid beta (Aβ) plaques but higher tau tangles density. No interaction effect was found for arteriolosclerosis. Women <75 years of age carrying the *APOE* ε4 allele had higher Aβ plaque burden than ε4 non‐carriers.

**DISCUSSION:**

Our results highlight the complex effect of sex on microvascular and AD‐associated pathologies in the MTL.

## Highlights

- Deep learning was used to obtain quantitative measures of proteinopathies.
- In the medial temporal lobe, women had fewer amyloid beta (Aβ) plaques and more tau tangles compared to men.
- Women with more cerebral amyloid angiopathy (CAA) had fewer Aβ plaques but a higher tau tangle density.
- After age stratification, women younger than 75 years and ε4 carriers had higher Aβ plaque burden.

## 1 INTRODUCTION

Globally, more women than men live with dementia (women‐to‐men ratio 1.69), and at least two thirds of patients living with Alzheimer's disease (AD) in the United States are women.^1^, ^2^ A growing body of epidemiological, clinical, and preclinical evidence suggests a variety of biological mechanisms at play in driving these sex differences, including genetic factors (sex chromosomes), sex hormones, transition into menopause, as well as differences in immune response.^3^ Sex differences are also reflected in the burden of the defining pathologies of AD,^4^, ^5^, ^6^, ^7^, ^8^, ^9^ which have been highlighted in whole‐brain histological studies, where women have been found to exhibit a higher burden of tau tangles and, to a lesser extent, amyloid beta (Aβ) plaques compared to men.^4^, ^5^, ^6^, ^7^, ^8^, ^9^

The effect of female sex on AD pathology may be further modified by age. In fact, some brain autopsy studies suggest that more women than men exhibit AD pathology^6^, ^7^ at or after age 75, although others reported higher burden of AD pathology in women independent of age.^7^, ^8^ Sex‐dependent effects on AD pathology burden might also be influenced by the apolipoprotein E (*APOE*) ε4 allele, the main genetic risk factor for late‐onset AD. In fact, *APOE* ε4 promotes seeding,^10^ fibrillar aggregation,^11^, ^12^ and reduced clearance of Aβ,^13^ promoting Aβ accumulation in parenchymal plaques and within the vessel wall of cerebral arterioles and capillaries (i.e., cerebral amyloid angiopathy [CAA]).^14^ Previous autopsy‐based studies have shown that women carrying ε4 present a higher burden of AD pathology,^4^, ^5^ although this finding was not replicated in other cohorts.^8^, ^9^ However, pooled data suggest that the detrimental effect of ε4 allele on women might be age dependent, with the highest risk before 75 years.^15^ These studies highlight the complexity of the inter‐relationship between sex, age, and *APOE* genotype.

Another factor that needs to be considered when investigating sex differences in AD is the presence of mixed pathologies beyond the AD‐defining proteinopathies, which is frequent in patients diagnosed with AD neuropathological changes (ADNC).^16^ For example, cytoplasmatic inclusions of phosphorylated transactive response DNA binding protein 43 (pTDP‐43) is a common and often co‐occurring pathology, which independently increases the risk of dementia.^17^ Another highly prevalent pathology in the aging brain is cerebral small vessel disease (CSVD), which often coexists with AD pathology,^18^, ^19^ independently contributes to dementia, and is also thought to exacerbate the burden of proteinopathies, possibly by impairing perivascular clearance, increasing blood–brain barrier permeability, and promoting neuroinflammation.^20^ The most common forms of sporadic CSVD are arteriolosclerosis—defined as thickening and hyalinosis of the vessel wall in the arterioles of the white matter—and CAA, that is, the vascular accumulation of Aβ in the leptomeningeal and cortical arterioles.

So far, the moderating effect of sex on the relationship between microvascular pathology (i.e., arteriolosclerosis and CAA) and AD‐associated proteinopathies (i.e., Aβ plaques, tau tangles, and pTDP‐43 cytoplasmatic inclusions) has not been well characterized. One reason is the difficulty of obtaining objective, quantitative, and continuous measures of pathological hallmarks and limited regional specificity of the pathological assessments. In this regard, a brain region that deserves specific attention is the medial temporal lobe (MTL), which is particularly vulnerable to early accumulation of AD pathology and to the co‐occurrence of microvascular changes and neurodegenerative proteinopathies such as Lewy body disease and pTDP‐43.^21^ Moreover, the MTL plays a crucial role in episodic memory, learning, and global cognition,^22^ which amplifies the clinical consequences of neurodegeneration within this brain region.^23^

Thus, a quantitative, region‐specific analysis of the relationship between AD‐associated proteinopathies and microvascular pathologies might hold important implications toward a better understanding of the role of sex in AD pathophysiology. In this study, we applied deep learning–based models to quantify hallmarks of AD and CSVD in histological sections of the MTL of brain donors with and without ADNC. We aimed to assess (1) the effect of sex on single AD‐associated proteinopathies in the MTL, while accounting for microvascular pathology and *APOE* genotype and (2) the interaction between sex and *APOE* genotype and between sex and microvascular pathology on AD‐associated proteinopathies.

## 2 METHODS

### 2.1 Donor selection

The present study included brain donors from the brain bank of the Massachusetts Alzheimer's Disease Research Center (MADRC), located at Massachusetts General Hospital (MGH) in Boston, Massachusetts, USA.^24^, ^25^ The brains were donated through the hospital's Memory Division Unit. To be included in the study, the following criteria had to be met: a comprehensive neuropathological assessment, diagnosis by a board‐certified neuropathologist, age of death of 50 years or older, and availability of well‐preserved histological and immunohistochemically stained sections from the MTL. Brain donors were excluded if they had a primary clinical or neuropathological diagnosis of frontotemporal dementia, dementia with Lewy bodies (DLB), Parkinson's disease (PD), large territorial infarcts, or inherited neurodegenerative disorders. Cases with a primary diagnosis of DLB were excluded to keep the focus on the ADNC rather than the PD continuum and to reduce the number of variables. Following this screening process in consecutive cases enrolled between January 1997 and November 2021, the final sample consisted of individuals with a primary neuropathological diagnosis of Alzheimer's disease neuropathological changes (or ADNC)^26^ and individuals without substantial neurodegenerative pathology, defined as not exceeding A1B0C1; however, vascular and neurodegenerative proteinopathies were allowed as co‐pathologies.^16^ For some of the donors, a diagnosis of dementia due to AD was determined during the clinical assessment according to the National Institute on Aging–Alzheimer's Association recommendations.^27^ Braak stage^28^ was determined by a board‐certified neuropathologist and groups were defined as follows: 0 = Braak stage 0 [control group]; 1 = Braak stages I/II; 2 = Braak stages III/IV; and 3 = Braak stages V/VI. Finally, Thal phase^29^ was also assessed in a subgroup of cases and then grouped as follows: 0 = Thal phase 0 [control group]; 1 = Thal phase 1/2; 2 = Thal‐phase 3; and 3 = Thal‐phase 4/5. All procedures were conducted in accordance with ethical guidelines established by the Partners Human Research Committee, the institutional review board for Massachusetts General Brigham.

**RESEARCH IN CONTEXT**

1. **Systematic review**: The burden of Alzheimer's disease (AD)–associated proteinopathies (amyloid beta [Αβ] plaques, tau tangles, and phosphorylated transactive response DNA binding protein 43 [pTDP‐43] cytoplasmatic inclusions) and cerebral small vessel disease (cerebral amyloid angiopathy [CAA] and arteriolosclerosis) differs between sexes. Furthermore, apolipoprotein E (*APOE*) has been suggested to have a modulating role on sex differences. However, the effect of sex on the pathological burden of the medial temporal lobe (MTL) and the interaction between sex and microvascular pathology on AD‐associated proteinopathies remain unknown.
2. **Interpretation**: Women with higher CAA burden showed lower Aβ plaques and higher tau tangle density in the MTL. *APOE* ε4 allele presence was associated with higher Aβ plaque burden in women <75 years, suggesting an age‐dependent interaction. These findings highlight the complex inter‐relationship between sex, AD‐associated proteinopathies, and microvascular pathology in the MTL.
3. **Future directions**: Future studies analyzing sex differences in AD pathology should address the effect of menopause, hormonal therapy, and vascular risk factors, alongside the role of α‐synuclein and limbic‐predominant age‐related TDP‐43 encephalopathy.

### 2.2 Histopathological work‐up

Sample blocks from pre‐defined regions of interests of the MTL (hippocampal body and entorhinal cortex) at the level of the lateral geniculate nucleus were obtained from formalin‐fixed (10%) coronal slabs and were then paraffin‐embedded in standard histological cassettes. The samples considered in this study included: (1) the anterior portion of the parahippocampal gyrus (perirhinal area/entorhinal cortex) and the amygdala; (2) the hippocampal body and posterior parahippocampal cortex. Subsequently, 7 µm–thick adjacent sections were cut on a microtome, stained for luxol‐fast‐blue with hematoxylin and eosin (LHE), and immunohistochemically stained for Aβ (monoclonal anti‐Aβ antibody, clone 6F/3D, 1:600, Dako, CAT#M087201‐2; antigen: Aβ‐peptide amino acids 8–17 and Aβ‐precursor protein; host species: mouse), phosphorylated tau (monoclonal anti‐pTau antibody, clone AT8, 1:5,000, Invitrogen, CAT #ENMN1020B; antigen: phosphorylated Tau [Ser202, Thr205]; host species: mouse), or pTDP‐43 (monoclonal anti‐pTDP‐43 antibody, clone 11‐9, 1:3000, CosmoBio, CAT #CAC‐TIP‐PTD‐M01A; antigen: TDP‐43 phosphorylated on serines 409 and 410; host species: mouse) with a Leica Bond RX autostainer. A summary of histological sections/regions of interest (ROIs) excluded from the analysis due to poor quality (tears, missing tissue areas) or due to suboptimal convolutional neural network (CNN) model performance is reported in Table S1. All sections were stained in house and then digitized using a NanoZoomer Digital Pathology (NDP)‐HT whole slide scanner (C9600‐12, Hamamatsu Photonics KK, Japan) at 20×, and the high‐resolution digital images were visualized using the NDP.view2 software (v 2.9.25).

### 2.3 Quantitative (AI‐based) and semi‐quantitative analysis of pathological hallmarks

**FIGURE 1:**
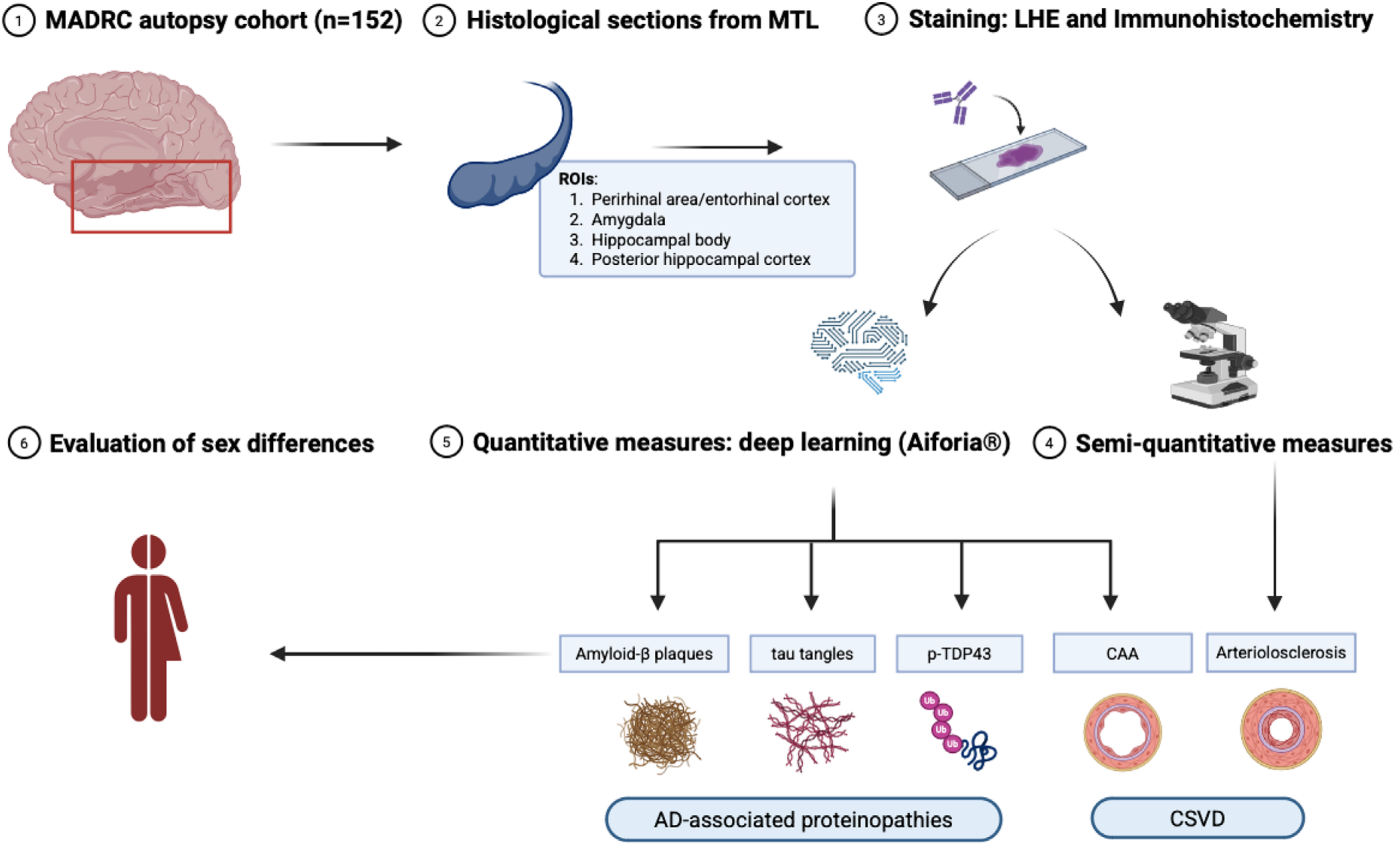
Study design overview. CAA, cerebral amyloid angiopathy; CSVD, cerebral small vessel disease; LHE, luxol‐fast‐blue with hematoxylin and eosin; MADRC, Massachusetts Alzheimer's Disease Research Center; MTL, medial temporal lobe; p‐TDP43, phosphorylated transactive response DNA binding protein‐43.

**FIGURE 2:**
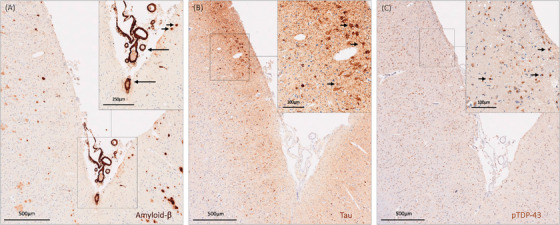
Example of medial temporal lobe histological sections exemplifying the co‐occurrence of AD‐associated proteinopathies and microvascular pathology in an autopsy case. Panel A: anti‐Aβ immunostained section showing the presence of Aβ plaques (inset panel, short arrows) and vessels with cerebral amyloid angiopathy (inset panel, arrow). Panel B: anti‐pTau immunostained section showing examples of tau tangles (inset panel, short arrows). Panel C: anti‐pTDP43 immunostained section showing neurons with pTDP‐43 cytoplasmatic inclusions (inset panel, short arrows). Aβ, amyloid beta; AD, Alzheimer's disease; p‐Tau, phosphorlyated Tau; p‐TDP‐43, phosphorylated transactive response DNA binding protein 43.

Microvascular pathology and AD‐associated proteinopathies were assessed across pre‐defined ROIs of the MTL: hippocampal body, posterior para‐hippocampal gyrus, rhinal cortex, and amygdala. Deep learning–based models (CNN) were built using the Aiforia platform and applied to obtain quantitative measures for percentage area of cortical CAA and Aβ plaques and density of both tau tangles and pTDP‐43 cytoplasmatic inclusions.^25^ A detailed description of the CNN training and validation methods can be found elsewhere^24^ and is here briefly summarized. Each model was trained on annotations made on a subset (≈10%) of digitized whole slides chosen to capture variability in image and staining quality across the dataset. The models consisted of multiple nested “layers” representing independent CNNs.^24^ Three AI models and the following derived measures were developed: (1) Aβ model: percentage area of cortical CAA (CAA area [mm^2^]/tissue area [mm^2^]) and percentage area of Aβ plaques (Aβ plaque area [mm^2^]/area [mm^2^]); (ii) tau model: density of tau tangles (number of tau tangles/tissue area [mm^2^]); (iii) pTDP‐43‐model: density of pTDP‐43 cytoplasmatic inclusions (number of pTDP‐43 cytoplasmatic inclusions/cortical tissue area [mm^2^]). Transfer learning was applied within Aiforia from a previously trained Aβ model to this new cohort. The artificial intelligence (AI)–based measures were validated against three independent human raters on 10% of the histological cohort, first comparing each rater to each CNN model and then, to evaluate the overall performance of the model, averaging all the human validators.^24^ All AI‐based models showed >80% precision and F1 score values overall and in comparisons with single raters,^25^ indicating that inter‐rater variability had minimal impact on the CNN performance. Moreover, the quantification of Aβ plaques was positively correlated with both Thal‐phase and semiquantitative score of severity, as reported previously.^24^, ^25^ Similarly, in previous works, we reported how CAA % area was positively correlated with the Vonsattel score and tau tangle density in the MTL with Braak stages.^24^, ^25^ Arteriolosclerosis severity was assessed in all arteries >15 µm diameter (to avoid inclusion of capillaries and pre‐capillary arteries) and <150 µm diameter on the LHE sections, using a previously described semiquantitative scale (0 = no arteriolosclerosis; 1 = mild arteriolosclerosis; 2 = moderate arteriolosclerosis; 3 = severe arteriolosclerosis),^30^ and a grade was assigned to each ROI, after calculating the proportion of vessels with each severity grade and their quantile distribution. Subsequently, arteriolosclerosis severity was dichotomized for each ROI in moderate/severe (i.e., grades 2/3) versus none/mild (i.e., grades 0/1), based on the quantile distribution. Furthermore, the median severity across ROIs was calculated to assess arteriolosclerosis at the subject level. A graphical overview of the study's methodological pipeline is presented in Figure 1, while histological examples of AD‐associated proteinopathies in the MTL are reported in Figure 2.

### 2.4 *APOE* genotyping

The *APOE* genotype of study donors was either obtained at MADRC via restriction fragment length polymorphism analysis^31^ or TaqMan polymerase chain reaction (PCR) assays (ThermoFisher Scientific, CAT#4351379, assays IDs: C___3084793_20 for rs429358 and C____904973_10 for rs7412)^32^ on genomic DNA purified from cerebellar frozen samples, or received from the National Centralized Repository for Alzheimer's Disease and Related Dementias (NCRAD). *APOE* genotype was then dichotomized in ε4 carrier (either ε4/– or ε4/ε4) versus non‐carrier for the analysis. Donors with a ε2/ε4 genotype were included in the ε4 carrier group.^33^

### 2.5 Statistical analysis

In univariate analyses, *t*‐test and chi‐square (or Fisher exact test) were used to calculate differences between sexes. Correlations among continuous variables were analyzed with Spearman's rank correlation test. Linear mixed‐effects (LME) models were then adopted, using the ROIs and Braak stage group as random effects (random intercept): the former variable was chosen to account for the regional distribution differences in proteinopathies across the MTL, whereas the latter accounted for disease severity in proteinopathies. Within‐subject variability was purposefully not included as a random effect, since any between‐subject differences related to sex would be absorbed into the subject‐level random intercept, thereby diluting any significant sex effect. The general model formula is as follows: Y*_ijk _* = β_0_ + β_1_X*_ijk_* + R1_0_ *_j_* + R2_0_ *_k_* + ε*_ijk_*_,_ where Y represents the specific AD‐associated proteinopathy value for subject *i* at the *j‐*th and *k*‐th level of the two random effects (ROIs and Braak stage, respectively); β_0_ and β_1_ the intercept and slope of the fixed effect X (sex); R1_0_ and R2_0_ the intercepts for the two random effects; and ε the residual error. Variables reporting percent areas and count density (i.e., CAA% area, Aβ plaques % area, tau tangle density, and pTDP‐43 inclusion density) were normalized using a square root transformation to address skewed distribution of the measures. Multivariable models were then built including a set of pre‐specified covariates of interest deemed relevant to control for confounders in sex associations (i.e., age at death, *APOE* genotype, AD‐associated proteinopathies, and microvascular pathology). The aim of this analysis was to evaluate the association of sex with the severity of single AD‐associated proteinopathies, while accounting for other pathologies. A sensitivity analysis removing donors with ε2/ε4 genotype was also performed to clarify their role in driving sex associations. In fact, in the context of late‐onset AD, ε2 allele represents the major neuroprotective variant, whereas the ε4 allele the major common risk variant.^33^ We then tested the interaction between sex and microvascular pathology (arteriolosclerosis and CAA% area) and between sex and *APOE* genotype on the burden of cortical Aβ plaques (%), tau tangles, and pTDP‐43 inclusion density, respectively. The latter interaction analysis was also stratified by age at death in three strata (i.e., age values <25th, between 25th and 75th, and >75th percentiles). This stratified approach aimed to explore interaction across age based on previous evidence, suggesting an age‐dependent effect of *APOE* genotype and sex.^15^ Finally, the Benjamini–Hochberg method was adopted to adjust for multiple comparisons. Statistical analyses were performed with R, version 4.4.0 (R Foundation for Statistical Computing, Vienna, Austria; www.R‐project.org) using the *lme4* package. Statistical significance was set at 0.05.

## 3 RESULTS

### 3.1 Cohort general characteristics

Overall, 604 brain donors were screened, 156 initially included and, after exclusion of 4 cases due to a secondary neuropathological diagnosis, 152 were analyzed in the present study (age at death 81.0 years [standard deviation ± 12.5], 58% female) (Figure S1).^25^ Baseline cohort details stratified by sex are summarized in Table 1. There was no significant age difference between sexes, and women had a higher nominal proportion of dementia diagnosis during life. *APOE* genotypes were balanced between sexes (Table S2) with *APOE* ε3 and ε2 carriers being, respectively, the most and least prevalent, and ε4 carriers representing ≈40%.^14^, ^34^, ^35^ Women had a slightly higher proportion of Braak stage III–IV, whereas no difference was found for Thal phase. However, we note that missing data (*n* = 63, 41%) for this variable could have masked the true effects.

**TABLE 1:** General characteristics of the study cohort (*n* = 152) according to sex.

Variable	Men (n = 63)	Women (n = 89)	*p* pairwise	*p* overall
Age at death (years), mean (SD)	79.8 (11.9)	81.8 (13.0)	–	0.3
Hemisphere analyzed (left), n (%)	29 (46%)	39 (44%)	–	0.7
Dementia diagnosis, n (%)	37 (60%)	57 (73%)	–	0.1
*APOE* alleles, n (%)				
ε2	9 (17%)	14 (18%)	1	
ε3	49 (91%)	72 (92%)	0.8	
ε4	20 (37%)	31 (40%)	0.9	
*APOE* ε4 alleles, n (%)				
ε4/–	17 (31%)	29 (37%)	0.6	0.6
ε4/ε4	3 (6%)	2 (3%)	0.6	
Braak tangle stage, n (%)				
0	7 (11%)	2 (2%)	0.13	**0.05**
I–II	15 (24%)	22 (25%)	1	
III–IV	17 (27%)	38 (43%)	0.14	
V–VI	24 (38%)	27 (30%)	0.5	
Thal phase, n (%)				
0–1	12 (26%)	9 (21%)	0.8	0.3
2–3	14 (30%)	8 (19%)	0.5	
4–5	21 (45%)	25 (60%)	0.5	
Aβ plaque % area, mean (SD)	1.03 (1.65)	0.59 (0.74)	–	*0.06*
tau tangle density, n/mm^2^, mean (SD)	14.7 (18.4)	15.8 (16.1)	–	0.7
pTDP‐43 inclusion density, n/mm^2^, mean (SD)	13.09 (28.47)	14.34 (16.93)	–	0.8
CAA% area, mean (SD)	0.16 (0.25)	0.07 (0.07)	–	**0.01**
Arteriolosclerosis severity, n (%)				
0 (none)	11 (18%)	18 (24%)	0.6	**0.01**
1 (mild)	41 (67%)	31 (40%)	**0.003**	
2 (moderate)	6 (10%)	17 (22%)	*0.09*	
3 (severe)	3 (5%)	11 (14%)	0.13	

*Note*: *t*‐test and chi‐square/Fisher's test were used to compare continuous and categorical variables, respectively. *p* values for pairwise comparisons are adjusted for multiplicity using the Benjamini–Hochberg correction. *p* values <0.05 are shown in bold, and *p* values between 0.05 and 0.10 are shown in italic.

Abbreviations: CAA, cerebral amyloid angiopathy; pTDP‐43, phopsphorylated transactive response DNA binding protein 43; SD, standard deviation.

### 3.2 Sex differences in AD‐associated proteinopathies and microvascular pathology

In univariate analysis (Table 1), women had lower Aβ plaque % area (*p* = 0.06), but no difference was evident for tau tangles or pTDP‐43 inclusion density. These results remained consistent when considering distribution across ROIs (Table 2). Fully adjusted models confirmed lower Aβ plaque % (*β* = –0.10, 95% confidence interval [CI_95%_] –0.20, –0.01; *p* = 0.035) and higher tau tangle density (*β* = 0.38, CI_95%_ 0.02, 0.75; *p* = 0.041) in women but no significant difference for pTDP‐43 inclusion density (Table 3). Sensitivity analyses removing *APOE* ε2/ε4 carriers substantially confirmed these results (Table S3), although the positive association between women and tau tangles was attenuated (*p* = 0.07). Women also showed overall higher nominal proportions of moderate and severe arteriolosclerosis compared to men (although not reaching statistical significance in adjusted pairwise comparisons), but a lower overall cortical CAA% area (mean % area 0.07% vs 0.16%, *p* = 0.01). These results were confirmed at the individual ROI level (Table 2), suggesting that these findings were not driven by regional distribution differences in microvascular pathology between sexes. Finally, when analyzing the correlation between AD‐associated proteinopathies and microvascular pathology, CAA—but not arteriolosclerosis—was positively associated with Aβ plaque % area and tau tangle density (Spearman's *ρ* = 0.7, *p* < 0.001 and *ρ* = 0.4, *p* < 0.001, respectively) (Figure S2), as we have reported previously in this cohort.^25^ Notably, these correlations were similar across both sexes.

**TABLE 2:** Summary measures for Alzheimer's disease‐associated proteinopathies and cerebral small vessel disease according to sex across ROIs (*n* = 608 sections).

	ROI	Men (n = 63)	Women (n = 89)	*p*‐value
Aβ plaque % area, mean (SD)	Hippocampal body	0.31 (0.55)	0.25 (0.35)	**<0.001**
	Posterior hippocampal cortex	1.78 (2.95)	1.13 (1.51)	
	Amygdala	0.89 (1.35)	0.39 (0.56)	
	Perirhinal area/entorhinal cortex	1.44 (2.38)	0.75 (1.14)	
tau tangle density, n/mm^2^, mean (SD)	Hippocampal body	9.09 (11.22)	11.39 (13.75)	0.6
	Posterior hippocampal cortex	13.20 (18.59)	16.60 (23.25)	
	Amygdala	19.70 (26.74)	16.43 (20.11)	
	Perirhinal area/entorhinal cortex	17.05 (19.79)	18.55 (18.67)	
pTDP‐43 inclusion density, n/mm^2^, mean (SD)	Hippocampal body	9.38 (24.06)	9.95 (14.14)	0.8
	Posterior hippocampal cortex	13.75 (34.13)	12.16 (16.78)	
	Amygdala	17.31 (34.42)	16.50 (21.28)	
	Perirhinal area/Entorhinal cortex	15.69 (31.13)	19.58 (27.54)	
CAA% area, mean (SD)	Hippocampal body	0.06 (0.10)	0.03 (0.05)	**<0.001**
	Posterior hippocampal cortex	0.26 (0.51)	0.12 (0.15)	
	Amygdala	0.08 (0.14)	0.03 (0.04)	
	Perirhinal area/Entorhinal cortex	0.24 (0.37)	0.09 (0.12)	
Arteriolosclerosis grade 2/3, n (%)	Hippocampal body	23 (37%)	40 (45%)	**0.002**
	Posterior hippocampal cortex	23 (37%)	39 (44%)	
	Amygdala	20 (32%)	43 (48%)	
	Perirhinal area/Entorhinal cortex	27 (43%)	46 (52%)	

*Note*: *p* values refer to mixed effect models analyzing the effect of sex (covariate) on specific Alzheimer's disease (AD‐associated proteinopathies and cerebral small vessel disease (CSVD)), using ROIs as random effects. *p* values <0.05 are shown in bold.

Abbreviations: Aβ, amyloid beta; CAA, cerebral amyloid angiopathy; pTDP‐43, phosphorylated transactive response DNA binding protein 43; ROI, region of interest; SD, standard deviation.

**TABLE 3:** Multivariable models for Aβ plaques, tau tangles, and pTDP‐43 inclusions across ROIs and Braak stage (random effects).

	Aβ plaques (% area)—sqrt			tau tangle density, n/mm^2^—sqrt			pTDP‐43 inclusion density, n/mm^2^—sqrt		
Predictors	*β* (SE)	95% CI	*p*	*β* (SE)	95% CI	*p*	*β* (SE)	95% CI	*p*
Age at death, y	0.03 (0.03)	−0.03; 0.08	0.3	−0.10 (0.11)	−0.32; 0.12	0.4	0.45 (0.17)	0.11; 0.79	**0.009**
Sex, women	−0.10 (0.05)	−0.20; –0.01	**0.035**	0.38 (0.19)	0.02; 0.75	**0.041**	0.27 (0.30)	−0.31; 0.85	0.4
*APOE* ε4 carrier	0.02 (0.05)	−0.08; 0.12	0.7	0.26 (0.19)	−0.10; 0.63	0.2	−0.04 (0.30)	−0.63; 0.54	0.9
Aβ plaque % area—sqrt	–	–	–	1.44 (0.19)	1.06; 1.81	**<0.001**	0.71 (0.33)	0.06; 1.36	**0.032**
tau tangle density, n/mm^2^—sqrt	0.11 (0.01)	0.08; 0.13	**<0.001**	–	–	–	0.08 (0.09)	−0.09; 0.25	0.3
pTDP‐43 inclusion density, n/mm^2^—sqrt	0.02 (0.01)	0.00; 0.04	**0.029**	0.04 (0.03)	−0.03; 0.11	0.2	–	–	–
CAA% area—sqrt	1.39 (0.13)	1.13; 1.65	**<0.001**	−0.18 (0.57)	−1.30; 0.94	0.7	0.40 (0.91)	−1.39; 2.20	0.7
Arteriolosclerosis, moderate/severe	0.03 (0.05)	−0.06; 0.13	0.5	0.06 (0.18)	−0.29; 0.41	0.7	−0.86 (0.28)	−1.32; –0.20	**0.008**
Observations/*R* ^2^	333/0.538			333/0.480			333/0.145		

*Note*: Only fixed effects are shown. The model formula is as follows: AD‐associated proteinopathy 1 ∼ Age‐at‐death + Sex + AD‐associated proteinopathy 2 + AD‐associated proteinopathy 3 + CAA + Arteriolosclerosis + (1|Braak stages) + (1|ROIs). *p* values <0.05 are shown in bold.

Abbreviations: Aβ, amyloid beta; CAA, cerebral amyloid angiopathy; CI, confidence interval; pTDP‐43, phosphorylated transactive response DNA binding protein 43; sqrt, square root transformed; SE, standard error.

### 3.3 Effect of the interaction between sex and CSVD on AD‐associated proteinopathies

**FIGURE 3:**
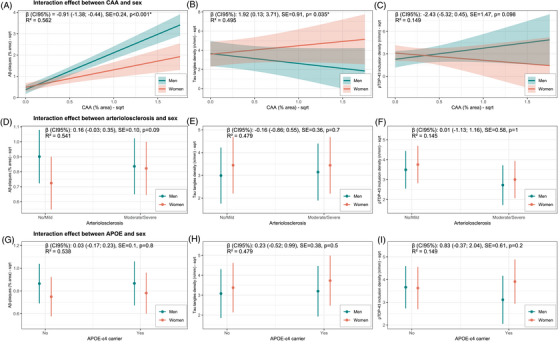
Predicted fixed‐effects outcomes for interaction of sex with microvascular pathology and *APOE*. Aβ, amyloid beta; CAA, cerebral amyloid angiopathy; pTDP‐43, phosphorylated transactive response DNA binding protein 43; SE, standard error.

In LME models with full covariate adjustment (i.e., age at death, *APOE* genotype, AD‐associated proteinopathies, and microvascular pathology), the interaction between sex and CAA revealed that women with higher CAA% area had lower Aβ plaque % area (*β* = –0.91, CI_95%_ –1.38, –0.44; *p* < 0.001), indicating that the positive association between parenchymal and vascular Aβ burden detected in the whole cohort was indeed reversed in women (Table 4, Figure 3). On the other hand, women with higher CAA% area showed higher tau tangle density (*β* = 1.92, CI_95%_ 0.31, 3.71; *p* = 0.035). Regarding arteriolosclerosis, we found a pattern of interactive association between female sex and Aβ plaque % area, although not reaching statistical significance (*β* = 0.16, CI_95%_ –0.03, 0.35; *p* = 0.09), suggesting that women with severe arteriolosclerosis might present a higher burden of Aβ plaques, whereas no effect was found for tau tangles. Similarly, no effect in the interaction of both microvascular pathologies (CAA/arteriolosclerosis) with sex was observed for pTDP‐43 cytoplasmatic inclusions. Overall, these results suggest an effect of female sex in mediating the association between microvascular pathology and AD‐associated pathology (i.e., Aβ plaque and tau tangles).

**TABLE 4:** Interaction analysis of sex with *APOE* genotype and microvascular pathology (i.e., cerebral amyloid angiopathy [CAA] and arteriolosclerosis) on Alzheimer's disease (AD)–associated proteinopathies across region of interest and Braak stage (random effects).

	Aβ plaques (% area)—sqrt			tau tangle density, n/mm^2^—sqrt			pTDP‐43 inclusion density, n/mm^2^—sqrt		
	*β* (SE)	95% CI	*p*	*β* (SE)	95% CI	*p*	*β* (SE)	95% CI	*p*
CAA interaction									
Sex, women	0.11 (0.07)	−0.04; 0.25	0.1	−0.07 (0.28)	−0.62; 0.49	0.8	0.82 (0.45)	−0.05; 1.70	*0.066*
Sex, women: CAA% area—sqrt	−0.91 (0.24)	−1.38; –0.44	<**0.001**	1.92 (0.91)	0.13; 3.71	**0.035**	−2.43 (1.47)	−5.32; 0.45	*0.098*
Observations/*R* ^2^	333/0.562			333/0.495			333/0.149		
Arteriolosclerosis interaction									
Sex, women	−0.18 (0.07)	0.31; –0.05	**0.007**	0.45 (0.25)	−0.03; 0.94	*0.07*	0.26 (0.40)	−0.51; 1.04	0.5
Sex, women: Arteriolosclerosis, moderate/severe	0.16 (0.10)	−0.03; 0.35	*0.09*	−0.16 (0.36)	−0.86; 0.55	0.7	0.01 (0.58)	−1.13; 1.16	1
Observations/*R* ^2^	333/0.541			333/0.479			333/0.145		
*APOE* interaction									
Sex, women	−0.12 (0.06)	−0.24; 0.01	***0.064***	0.30 (0.23)	−0.16; 0.76	0.2	−0.03 (0.37)	−0.76; 0.70	0.9
Sex, women: *APOE* ε4 carrier	0.03 (0.10)	−0.17; 0.23	0.8	0.23 (0.38)	−0.52; 0.99	0.5	0.83 (0.61)	−0.37; 2.04	0.2
Observations/*R* ^2^	333/0.538			333/0.479			333/0.149		

*Note*: Each model is adjusted for age, *APOE* ε4 allele, AD‐associated proteinopathies (i.e., those not included as dependent variables), microvascular pathology (CAA and arteriolosclerosis). For each model, only sex and the interaction term are shown. *p* values <0.05 are shown in bold, and *p* values between 0.05 and 0.10 are shown in italic.

Abbreviations: Aβ, amyloid beta; CAA, cerebral amyloid angiopathy; pTDP‐43, phosphorylated transactive response DNA binding protein 43; sqrt, square root transformed; SE, standard error.

### 3.4 Effect of the interaction between sex and *APOE* genotype on AD‐associated proteinopathies

No overall effect of the interaction between sex and *APOE* genotype on AD‐associated proteinopathies emerged (Table 4). However, in age‐stratified analyses (Table 5, Figure S3), a positive interaction between female sex and *APOE* ε4 allele on Aβ‐plaque % area was observed in the lowest age group (<75 years, *β* = 0.54, CI_95%_ 0.13, 0.95; *p* = 0.01), whereas no interaction was found for cases in the age groups between 75 and 90 years, with a decreasing effect size for increasing age. Women <75 years old had a higher burden of tau tangles (*β* = 2.08, CI_95%_ 1.00, 3.15; *p* < 0.001) and pTDP‐43 inclusions (*β* = 1.33, CI_95%_ 0.06, 2.60; *p* = 0.041) compared to men of the same age‐group, but no significant effect was observed for the sex × *APOE* ε4 interaction on tau tangle and pTDP‐43 inclusion densities in the youngest age group. However, the interaction between female sex and *APOE* ε4 was significant for pTDP‐43 inclusion density for cases in the age group spanning 75–90 years (*β* = 2.43, CI_95%_ 0.65, 4.20; *p* = 0.008).

**TABLE 5:** Age‐stratified interaction analysis for sex and *APOE* genotype, defined according to the percentiles of age distribution. For each model only the estimate for sex and interaction term are shown.

	Aβ plaques (% area)—sqrt			tau tangle density, n/mm^2^—sqrt			pTDP‐43 inclusion density, n/mm^2^—sqrt		
Sex‐*APOE* interaction	*β* (SE)	95% CI	*p*	*β* (SE)	95% CI	*p*	*β* (SE)	95% CI	*p*
<75 years									
Sex, women	−0.38 (0.14)	−0.66; –0.09	**0.010**	2.08 (0.54)	1.00; 3.15	**<0.001**	1.33 (0.64)	0.06; 2.60	**0.041**
Sex, women: *APOE* ε4 carrier	0.54 (0.21)	0.13; 0.95	**0.01**	−1.82 (0.79)	−3.38; –0.25	**0.024**	0.59 (0.94)	−1.28; 2.46	0.5
Observations/*R* ^2^	82/0.645			82/0.689			82/0.385		
75–90 years									
Sex, women	−0.27 (0.10)	−0.46; –0.09	**0.005**	0.42 (0.39)	−0.36; 1.20	0.3	−1.87 (0.61)	−3.08; –0.67	**0.002**
Sex, women: *APOE* ε4 carrier	0.11 (0.14)	−0.17; 0.39	0.4	0.10 (0.58)	−1.04; 1.25	0.9	2.43 (0.90)	0.65; 4.20	**0.008**
Observations/*R* ^2^	174/0.620			174/0.448			174/0.299		
>90 years									
Sex, women	0.10 (0.11)	−0.11; 0.32	0.4	−0.41 (0.41)	−1.23; 0.40	0.3	1.32 (0.67)	−0.02; 2.67	*0.054*
Sex, women: *APOE* ε4 carrier	−0.42 (0.25)	−0.91; 0.08	***0.097***	1.38 (0.95)	−0.52; 3.28	*0.15*	−1.42 (1.61)	−4.64; 1.79	0.4
Observations/*R* ^2^	77/0.564			77/0.413			0.366		

*Note*: Each model is adjusted for age, *APOE* ε4 genotype, AD‐associated proteinopathies (i.e., those not included as dependent variables), and microvascular pathology (cerebral amyloid angiopathy and arteriolosclerosis). *p* values <0.05 are shown in bold, *p* values between 0.05 and 0.10 are shown in italic.

Abbreviations: Aβ, amyloid beta; AD, Alzheimer's disease; *APOE*, apolipoprotein E; CI, confidence interval; pTDP‐43, phosphorylated transactive response DNA binding protein 43; SE, standard error; sqrt, square root transformed.

## 4 DISCUSSION

This autopsy study suggests an effect of sex with both AD‐associated proteinopathies and microvascular pathology in the MTL, using data from an autopsy cohort of donors with and without ADNC. Our results confirm and expand previous neuropathological findings^4^, ^5^, ^6^, ^7^, ^8^, ^9^ regarding sex differences in AD and microvascular pathology. Specifically, we found that tau tangle density was higher in women compared to men, which became evident when adjusting for concomitant AD‐associated and microvascular pathologies. In particular, the association between women and higher tau tangle density was significant when adjusting for Aβ plaque density, suggesting that, for any given level of Aβ plaque, women present higher levels of tau tangles in the MTL. These results are in line with the hypothesis that the higher tau burden found in women compared to men is magnified in the context of high neocortical Aβ deposits, as suggested in a recent analysis of the Wisconsin Registry for Alzheimer's Prevention Positron Emission Tomography study.^36^ More generally, these findings should be interpreted in the context of the growing body of evidence suggesting a synergistic relationship between Aβ and tau,^37^, ^38^, ^39^, ^40^, ^41^ where the presence of Aβ deposition in neocortical regions seems to precede and exacerbate tau accumulation in the MTL and increases its likelihood of spreading to neocortical brain areas.^42^

Contrary to previous reports,^4^, ^5^, ^6^, ^7^, ^8^, ^9^ we found a negative association of female sex with Aβ plaque percentage area. However, previous studies assessed Aβ pathology globally, whereas our result might be specific of the MTL. Furthermore, the age‐dependent distribution of AD pathology across sexes might have influenced this finding.^5^ In fact, available neuropathological evidence shows that although women generally have a higher burden of global AD pathology (calculated as an averaged summary of neuritic or diffuse plaques and neurofibrillary tangle counts) compared to men, older women presented lower global AD pathology and this result was driven by lower Aβ plaques.^8^ Moreover, we noted that previous studies did not account systematically for the concomitant co‐pathologies in the analysis of sex differences, suggesting that the lower burden of Aβ plaques may also reflect the adjustment for previously unmeasured confounders.^5^, ^8^ Of interest, the low explanatory power of models exploring pTDP‐43 inclusion density suggests that factors other than sex, Aβ, tau, and microvascular pathology might be more relevant in determining the levels of this AD‐associated proteinopathy. On a broader perspective, these results support the concept that sex differences in the context of AD pathology should be approached considering the joint distribution of related co‐pathologies.

These findings provide further insights into the effects of sex differences on the severity of microvascular pathology in the MTL. Previous data from the Religious Orders Study and the Rush Memory and Aging Project showed that women had a higher frequency of severe arteriolosclerosis independent of age at death.^8^ Another study focusing on an autopsy cohort of clinical cases with CAA did not find a clear difference between sexes for this microvascular pathology, although men showed a higher proportion of cortical superficial siderosis—a characteristic hemorrhagic manifestation of severe CAA^43^—and cortical iron burden compared to women.^44^ In our study, we confirmed that previous findings of a higher burden of arteriolosclerosis in women, independent of age, also apply to the MTL.^8^ However, we did not adjust for vascular risk factors during life. It is therefore plausible that this association might partially reflect a selection bias, as in existing neuropathological cohorts women reaching older age have generally more cardiovascular risk factors compared to men.^8^ The presence of vascular risk factors has been consistently associated with a higher incidence of cardiovascular events in women compared to men,^45^ so this relationship seems more complex and likely influenced by the estrogen level decreases after menopause and, more in general, by the differential vascular aging trajectories between the sexes.^46^ Furthermore, we observed a lower burden of cortical CAA in women compared to men, although former reports analyzed global and not regional CAA burden.^44^ In this regard, clinical data showing that men with CAA have earlier disease onset, and a higher burden of lobar cerebral microbleeds seem to support our results.^47^

Our findings also provide initial evidence for an interactive effect of female sex and CSVD on AD pathology. Specifically, the interaction found between sex and CAA on tau tangles (Table 4) highlights the differential resilience to AD pathology across the sexes for increasing levels of microvascular pathology. In fact, the higher tau tangle density observed in women, with higher CAA burden compared to men, might point to a sex‐specific tau clearance impairment as a potential unifying mechanism to explain this finding. On the other hand, the negative interaction observed between sex and CAA on Aβ plaques is less clear to interpret in this context but might simply reflect the already observed reduced Aβ plaque burden compared to men and the regional specificity of our analysis.

Our results also confirm previous evidence suggesting a positive interactive effect between the *APOE* ε4 allele and female sex for younger individuals.^15^ In fact, our findings support the hypothesis that *APOE* ε4 genotype significantly drives the accumulation of AD pathology in women in the lower age groups (<75 years). Although this finding might be partly reflective of a selection bias toward a more severe AD pathology in younger women donors, these results are in line with a meta‐analysis pooling clinical data from 27 independent research cohorts showing a susceptibility window for *APOE* effect on sex between 65 and 75 years. Therefore, our findings corroborate the hypothesis that *APOE* may modulate the effect of sex on AD pathology early in the disease history,^15^ possibly by magnifying the negative effects of post‐menopausal hormonal changes on vascular health and dementia risk in women.^3^, ^48^

Taken together, our results highlight the complex network of sex‐driven associations between AD‐associated proteinopathies and microvascular pathology in the MTL with potential clinical implications. Indeed, a clearer pathological understanding of sex differences might be relevant in the context of the new anti‐amyloid immunotherapies. In fact, a subgroup analysis from the Lecanemab Clarity AD trial showed that the beneficial effect observed on cognitive and functional outcomes consistently favored men over women, highlighting a sex‐specific effect in therapeutic response.^49^ Given the known strong relationship between tau burden and cognitive impairment, our finding of a higher tau tangle density in women, even after age adjustment, could explain—at least partially—these clinical findings. The specificity of our results to the MTL has additional relevance when considering the accumulation of tau (and less so Aβ) in this brain region early during the disease course^50^ and the central role of the MTL in global cognition and memory.^22^ Moreover, as therapeutic approaches targeting *APOE* are being tested,^14^ the age‐dependent sex interaction found in our study, if replicated, might hold clinical relevance. However, whether these observed neuropathological differences could translate into a more personalized and effective therapeutic approach will require further clinical–pathological studies.

Our study has several limitations. First, data regarding vascular risk factors, neuropsychological testing, and neuroimaging during life were not available, thereby limiting clinical‐pathological correlations. In this regard, future studies should address the role of age at menopause and its characteristics (i.e., surgical vs natural) and exposure to hormonal replacement therapy, to better characterize differences driven by estrogen exposure, as shown previously.^51^, ^52^ Among these factors, sex hormones have been accredited a central role in AD due to their established neuroprotective effect. In fact, population‐level data support that a lower age at natural menopause and lower reproductive spans (both proxies of a lower lifetime exposure to estrogens) were associated with increased risk of dementia.^53^ Second, it is plausible that our findings might not be applicable outside the MTL, thereby limiting their external generalizability. However, we note that our study aimed at analyzing specifically the MTL given its role as a neuropathological hub in AD, and therefore provided relevant evidence in the understanding of sex differences in AD‐associated neuropathologies. Third, the limited sample size of some age strata in analyses exploring the interaction between *APOE* and sex might have masked true differences. Fourth, we did not examine sex differences in Lewy body pathology, which is known to occur more frequently in men than in women. Future studies should therefore investigate this established sex difference and its interaction with microvascular pathology in the MTL. Finally, although it is plausible that our study might be affected by some degree of selection bias, there is no evidence for a systematic sex difference in referral patterns for brain donation in other cohorts.^54^ These limitations notwithstanding, the study's main strength is the quantitative AI‐driven method used to obtain continuous and objective pathological measures. This approach enabled the implementation of more complex statistical models, and therefore allowed us to account for concomitant co‐pathologies across ROIs and to address potential confounders that were not considered in previous studies. In this regard, it is worth noticing that the semi‐quantitative nature of the arteriolosclerosis assessment and its categorical classification might have masked true effects.

## 5 CONCLUSIONS

To summarize, our study quantifies the effect of sex on AD‐associated and microvascular pathology in the MTL of a cohort of brain donors with and without ADNC. We found that women have higher regional tau tangle density and lower Aβ plaque burden. However, the latter effect was partially dependent on *APOE* genotype, as women <75 years and carrying *APOE* ε4 exhibited higher levels of Aβ plaques. Women also had a higher burden of arteriolosclerosis (independent of age), but lower burden of cortical CAA, compared to men. These results highlight the complex crosslinks of sex differences among pathologies in the MTL and hold potential implications for the understanding of sex‐specific response to disease‐modifying therapies againstAD.

## CONFLICT OF INTEREST STATEMENT

The authors declare no conflict of interest relevant to the manuscript.

## CONSENT STATEMENT

All donors or their next of kin gave written informed consent for brain donation, and the study received approval from the Massachusetts General Hospital Institutional Review Board.

## Supporting information



Supporting Information

Supporting Information
