# Supplementary material for: APOE‐mediated sex differences in microvascular pathology and AD‐associated proteinopathies in the medial temporal lobe
Source: Alzheimers Dement. 2026 Mar 14;22(3):e71206. doi: 10.1002/alz.71206 (PMC13093349; doi:10.1002/alz.71206)
Supplement: Supplementary file 1 — Supporting Information [file ALZ-22-e71206-s002.docx]

**SUPPLEMENTARY TABLES**

**Table S1:** Missing values due to low quality of the histological section or suboptimal CNN model performance

| **Variable** | **ROI** | **Missing, No (%)** | | |
| --- | --- | --- | --- | --- |
|  |  | **Overall** | Men | Women |
| Aβ plaque | Hippocampal body | 37 (24%) | 13 (20%) | 24 (26%) |
|  | Posterior hippocampal cortex | 30 (19%) | 12 (19%) | 18 (20%) |
|  | Amygdala | 24 (15%) | 10 (15%) | 14 (15%) |
|  | Perirhinal area/Entorhinal cortex | 26 (17%) | 11 (17%) | 15 (16%) |
| Tangle density | Hippocampal body | 20 (13%) | 5 (7%) | 15 (16%) |
|  | Posterior hippocampal cortex | 19 (12%) | 6 (9%) | 13 (14%) |
|  | Amygdala | 15 (9%) | 6 (9%) | 9 (10%) |
|  | Perirhinal area/Entorhinal cortex | 19 (12%) | 7 (11%) | 12 (13%) |
| pTDP-43 inclusions | Hippocampal body | 19 (12%) | 8 (12%) | 11 (12%) |
|  | Posterior hippocampal cortex | 17 (11%) | 8 (12%) | 9 (10%) |
|  | Amygdala | 19 (12%) | 10 (15%) | 9 (10%) |
|  | Perirhinal area/Entorhinal cortex | 19 (12%) | 9 (14%) | 10 (11%) |
| CAA | Hippocampal body | 33 (21%) | 12 (19%) | 21 (23%) |
|  | Posterior hippocampal cortex | 29 (19%) | 12 (19%) | 17 (19%) |
|  | Amygdala | 23 (15%) | 9 (14%) | 14 (15%) |
|  | Perirhinal area/Entorhinal cortex | 24 (15%) | 10 (15%) | 14 (15%) |
| Arteriolosclerosis | Hippocampal body | 7 (4%) | 0 (0%) | 7 (7%) |
|  | Posterior hippocampal cortex | 6 (3%) | 0 (0%) | 6 (6%) |
|  | Amygdala | 5 (3%) | 2 (3%) | 3 (3%) |
|  | Perirhinal area/Entorhinal cortex | 8 (5%) | 2 (3%) | 6 (6%) |

**Legend:** CNN=Convolutional Neural Network; CAA=cerebral amyloid angiopathy

**Table S2:** *APOE* genotypes overall and according to sex

| ***APOE* genotype, No. (%)** | **Overall n=152** | **Men n=63** | **Women n=89** |
| --- | --- | --- | --- |
| ε2−ε2 | 1 (1%) | 0 (0%) | 1 (1%) |
| ε2−ε3 | 17 (13%) | 7 (13%) | 10 (13%) |
| ε2−ε4 | 5 (4%) | 2 (4%) | 3 (4%) |
| ε3−ε3 | 63 (48%) | 27 (50%) | 36 (46%) |
| ε3−ε4 | 41 (31%) | 15 (28%) | 26 (33%) |
| ε4−ε4 | 5 (4%) | 3 (6%) | 2 (3%) |

**Legend:** missing values n=20 (13%, men 9, women 11)

|  | **Aβ-plaques (% area) –**  **sqrt** | | | **Tau tangles density (n/mm^2^) –**  **sqrt** | | | **pTDP-43 inclusion density (n/mm^2^) - sqrt** | | |
| --- | --- | --- | --- | --- | --- | --- | --- | --- | --- |
| **Predictors** | **β (SE)** | **95% CI** | **p** | **β (SE)** | **95% CI** | **p** | **β (SE)** | **95% CI** | **p** |
| Age at death (y) | 0.02 (0.03) | -0.03; 0.08 | 0.4 | -0.12 (0.11) | -0.33; 0.09 | 0.3 | 0.42 0.16) | 0.09; 0.74 | **0.012** |
| Sex (women) | -0.11 (0.05) | -0.21; -0.01 | **0.033** | 0.35 (0.19) | -0.03; 0.72 | ***0.07*** | 0.21 (0.31) | -0.39; 0.82 | 0.5 |
| *APOE-*ε4 carrier | 0.05 (0.05) | -0.05; 0.15 | 0.3 | 0.18 (0.19) | -0.20; 0.55 | 0.4 | 0.08 (0.31) | -0.53; 0.69 | 0.8 |
| Aβ-plaque % area - sqrt | - | - | **-** | 1.49 (0.19) | 1.11; 1.87 | **<0.001** | 0.60 (0.34) | -0.07; 1.26 | *0.078* |
| Tangle density (n/mm^2^) - sqrt | 0.11 (0.01) | 0.09; 0.14 | **<0.001** | - | - | **-** | 0.11 (0.09) | -0.06; 0.29 | 0.2 |
| pTDP-43 inclusions density (n/mm^2^) - sqrt | 0.02 (0.01) | -0.00; 0.03 | ***0.087*** | 0.05 (0.03) | -0.02; 0.12 | 0.15 | - | - | **-** |
| CAA % area - sqrt | 1.41 (0.13) | 1.15; 1.67 | **<0.001** | -0.40 (0.58) | -1.54; 0.74 | **0.5** | 0.62 (0.93) | -1.22; 2.45 | 0.5 |
| Arteriolosclerosis (moderate/severe) | 0.04 (0.05) | -0.05; 0.14 | **0.4** | -0.00 (0.18) | -0.36; 0.35 | **1** | -0.76 (0.29) | -1.34; -0.19 | **0.009** |
| **Observations / R^2^** | 320 / 0.549 | | | 320 / 0.505 | | | 320 / 0.161 | | |

**Table S3:** Sensitivity analysis removing cases with *APOE*-ε2/ε4 genotype

**Legend:** sqrt = square root transformed. SE = standard error. Only fixed effects are shown.

**SUPPLEMENTARY FIGURES**

**Figure S1:** Study population flowchart. MADRC = Massachusetts Alzheimer’s Disease Research Center; MTL= medial temporal lobe; ADNC = Alzheimer’s disease neuropathological changes.

**Figure S2:** Correlation matrices showing Spearman correlation coefficients of univariate correlations across AD-associated proteinopathies, microvascular pathologies (i.e. CAA % area and arteriolosclerosis severity expressed as grade 0, 1, 2, and 3), *APOE* genotype (coded as 0 = no ε4 alleles, 1 = ε4/-, and 2 = ε4/ε4), and Braak stage. Values represent means across ROIs in each subject. Panel A shows correlations overall (i.e. independent of sex), while panel B and C show correlations for women and men only respectively. *p<0.05; **p<0.01; ***p<0.001.

^^

**Figure S3:** Predicted fixed-effect outcomes for interaction of sex with APOE across age strata.
